# A Brief Review on Flexible Electronics for IoT: Solutions for Sustainability and New Perspectives for Designers

**DOI:** 10.3390/s23115264

**Published:** 2023-06-01

**Authors:** Graziella Scandurra, Antonella Arena, Carmine Ciofi

**Affiliations:** Department of Engineering, University of Messina, 98166 Messina, Italy; arenaa@unime.it (A.A.); cciofi@unime.it (C.C.)

**Keywords:** flexible electronics, sustainable electronics, green electronics, energy harvesting, IoT, designer’s perspective, simulation tools, flexible devices characterization

## Abstract

The Internet of Things (IoT) is gaining more and more popularity and it is establishing itself in all areas, from industry to everyday life. Given its pervasiveness and considering the problems that afflict today’s world, that must be carefully monitored and addressed to guarantee a future for the new generations, the sustainability of technological solutions must be a focal point in the activities of researchers in the field. Many of these solutions are based on flexible, printed or wearable electronics. The choice of materials therefore becomes fundamental, just as it is crucial to provide the necessary power supply in a green way. In this paper we want to analyze the state of the art of flexible electronics for the IoT, paying particular attention to the issue of sustainability. Furthermore, considerations will be made on how the skills required for the designers of such flexible circuits, the features required to the new design tools and the characterization of electronic circuits are changing.

## 1. Introduction

The term sustainability has now become commonly used, it is of great importance and is also used in different contexts. It was used for the first time in 1992, during the first UN Conference on the environment. The definition of sustainability that has been given is this: *Condition of a development model capable of ensuring the satisfaction of the needs of the present generation without compromising the possibility of future generations to realize their own* [1]. This definition is centered not only on the economy and society, but above all on ecology. Sustainability and sustainable development are linked to a new idea of well-being that takes into account people’s quality of life. Environmental sustainability requires responsibility in the use of resources. It is therefore a development model to which everyone can and must contribute, starting from the awareness that every action performed by each of us has a deep impact on the environment.

**Figure 1:**
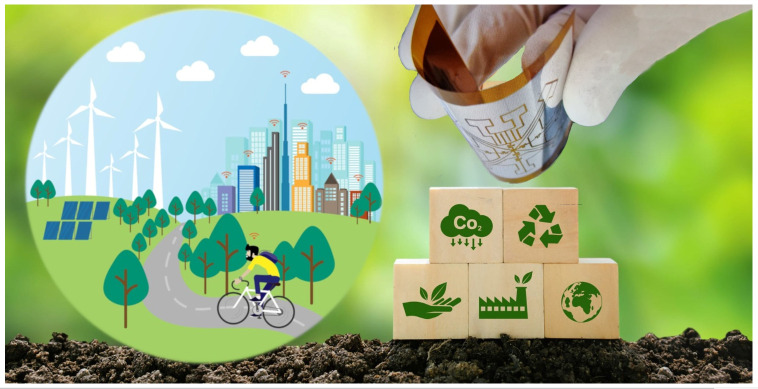
Flexible electronics is an important building block for the creation of a sustainable and interconnected world.

Based on these considerations, the world of electronics, which for decades has been increasingly pervasive in all sectors of life (industry, medical, automation, automotive, military, consumption), cannot fail to pay maximum attention to the issue of sustainability. The electronics as fuel of the Internet of Things technology is surely leading us in a new way of conducting our lives and cities [2], also allowing the optimization of the production processes of companies and industries and the management of services and infrastructures, limiting the consumption of resources and pollution. Management of public lighting [3,4,5,6,7], air quality [8,9,10,11,12,13,14,15,16,17,18,19,20,21,22] and noise pollution monitoring [23,24,25,26,27,28,29], smart home [30,31,32,33,34,35,36,37,38,39,40,41,42,43], smart roads, smart cars, urban mobility and transport [44,45,46,47,48,49,50,51,52,53,54,55,56,57,58,59,60,61,62,63], food and agriculture [64,65,66,67,68,69,70,71,72,73,74,75,76,77,78,79,80,81,82,83], smart factories [84,85,86,87,88,89,90,91] and medicine [92,93,94,95,96,97,98,99,100,101,102] are examples of the great potentialities of the IoT. However, the increase in connectivity inevitably translates into an increase in electronic devices and systems (sensors, data acquisition and processing systems, communication systems) and therefore the problem of respecting the environment, both in the production step and disposal of disused systems is, nowadays, of fundamental importance also in the field of the IoT industry. Thanks to the availability of eco-compatible materials, flexible electronics, which is a solution that is increasingly gaining space in many applications due to its portability, wearability and low cost, could be the right path towards an increasingly green IoT (Figure 1).

**Figure 2:**
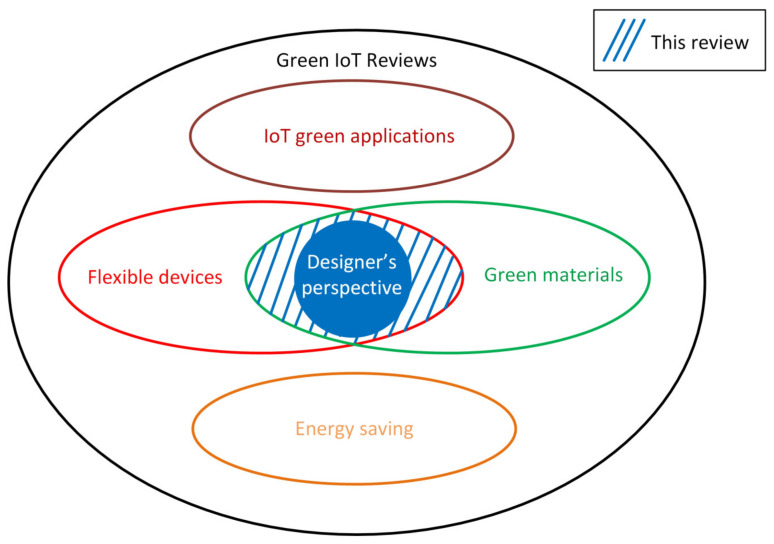
Collocation of this paper with respect to other review papers on “green IoT”. In this work we focused on solutions for IoT that are contextually flexible and eco-friendly, and we want to highlight how the skills of electronic circuit designers and the features of simulation and CAD (computer aided design) software are changing to accomplish modern IoT systems designs.

Although there are many works in the scientific literature on IoT or on flexible implementations of IoT technology, there is still no overview focused exclusively on solutions that are contextually flexible and green. The most considered green aspects so far for the IoT are those aimed at energy saving [103]. In Figure 2 the collocation of this work in the available literature is explained.

**Figure 3:**
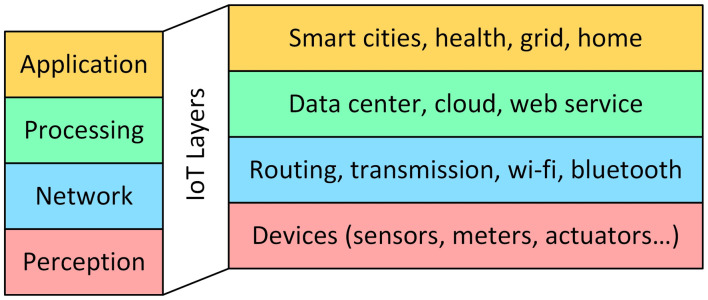
Typical architecture of an IoT system. In this figure the four principal layers are shown, but the more complex IoT architectures may have other further layers.

To better understand, let’s consider, as they are represented in Figure 3, the main layers of an IoT architecture [104]. The perception layer (or devices layer) consists of several devices—sensors, cameras, actuators, memories, RFID that sense, acquire, store, display data and perform tasks. The network layer transmits data from devices to an on-premises or cloud data center. The processing layer (or middleware layer), which typically leverages many connected computers simultaneously, performs cloud computing, storage, networking, and security performance. The application layer decodes and compiles data in forms that are easy for users to understand, such as graphs and tables. Programs for device control and monitoring, as well as process control software, are typical examples of the application layer of IoT architecture. The more complex IoT architectures have three further layers, not shown in the figure, i.e., edge, business and security.

In this review we describe the prospects of the key technologies of flexible electronics, such as RFIDs, sensors, memories and energy harvesters, that are the basis of the perception layer of an IoT architecture, highlighting the solutions that make it possible to achieve the goal of sustainability. First, in the next section, we will illustrate solutions to make the substrates of various devices in a sustainable way, because the choice of a green substrate is a key factor that is common to all devices. In conclusion, a discussion on how the skills of an electronic circuit designer, the features of the simulation and design tools and the characterization of produced devices must change, will be performed.

## 2. Green Substrates: Paper and “Nanopaper”

The choice of the substrate on which to make a flexible device is surely a key factor for sustainable IoT because the greater quantity of material that makes up the device is precisely the substrate [105]. 

With the advent of flexible electronics, the favored substrates on which to build devices have, for a long time, been plastic materials. However, discarded plastics degrade to form micro and nano-plastics that are hazardous to human beings and the environment. If one thinks of the implementation of flexible devices that are “green”, surely paper is the first material that comes to mind as a substrate to substitute plastic [106]. In fact, paper is widely and easily available, low-cost, recyclable and biodegradable. Table 1 shows a comparison between paper and the plastic materials mostly used as substrates, in terms of impacts on climate change and resource use [107]. In this regard, it may be useful to recall that studies conducted on these same indicators as regards the production of silicon, the fundamental semiconductor in the electronics industry, have highlighted a development in the wrong direction for the silicon industry, facing increasing climate related pressures [108]. 

Although it is very promising from an environmental point of view and several devices made on paper substrates have appeared in the last decade, the use of paper as a substrate is still limited, due to the high surface roughness and poor barrier properties against water and solvents [106]. However, if we consider that in applications in the IoT field, and therefore in electronics, one of the main properties of the substrates is that of allowing optimization of the device performance in terms of conductivity, paper substrates have performances no lower than the plastic ones most used up to now. In [106] an interesting comparison is made between different paper substrates and PET substrates. The main results are summarized in Table 2.

Continuing with the comparison between paper and plastic substrates, wishing to evaluate the performances in terms of elasticity, in Table 3 we report Young’s modulus. Among the plastic materials we have considered PET, precisely because it is the most used, PEN (polyethylene naphthalate) which has performances in terms of elasticity superior to other plastic substrates, and PDMS (polydimethylsiloxane), a popular elastomer in the manufacture of stretchable devices [109]. Results in Table 3 show that paper substrates can offer elastic performances comparable to PDMS under proper coating conditions.

Obviously, if the goal of making flexible devices that are absolutely sustainable is to be achieved, the separation of electronic materials, conductive metallic inks in most cases, from the paper substrate at the end of life of the devices must be easily performed. To overcome these limitations, a few solutions based on coating approaches have been presented to improve paper substrate performances. As an example, the use of shellac, that is a cheap biopolymer, has been proposed in [110]. Shellac, employed as a coating surface for paper substrates, forms planarized, printable surfaces. At the end of the life of the device, shellac behaves as a sacrificial layer that can be removed by immersing the printed device in methanol, enabling the separation of the paper substrate. Nevertheless, coating procedures and other surface treatments are not effective for all electronics applications [111,112]. In the last period, “nanopaper”, that is, planar substrates made of cellulose nanomaterials (CNM), gained relevance [113,114,115,116,117,118,119,120]. CNM are nanosized particles with highly ordered cellulose chains aligned along the bundle axis, that exhibit interesting characteristics with respect to pulp fibers and wood particles, such as high mechanical properties, low thermal expansion, low density, and simplicity of treatment that allows the implementation of additional functionalities [121,122,123]. To focus on sustainability, it is also important to evaluate the end-of-life performance, that is to carry out a study on the biodegradability of materials. In [114], for example, a comparison between the biodegradation of CNM samples with respect to microcrystalline cellulose (MCC), and a commercial thermoplastic polyurethane (TPU) has been performed and the results are summarized in Table 4.

The results reported in [114] show that in the first 70 days of testing, the biodegradability rate of the CNF-HEC compounds is comparable to that of pure cellulose, while subsequently there is a slowdown. Although there is no doubt that the biodegradability of cellulose-based samples is far superior to that of plastic materials, it is certainly clear that, to further improve the state of the art, studies need to be conducted to understand how to optimize the performance of paper substrates without lowering the biodegradability performance too much compared to pure cellulose. The biodegradability of the printed substrate is slightly lower than that of the non-printed substrate, also highlighting the importance of working on the eco-sustainability of the conductive layers. Without any doubt, the “nanopaper” technology, that is a relatively low-cost technology [124,125,126] for substrate fabrication for IoT applications, is strategic to fuel a transition toward a sustainable and green IoT, also working on the use of optimized nanocellulose with other materials and hybrid structures [127,128,129,130,131]. 

## 3. Perception Layer Devices

The main perception layer devices that will be considered in the following, due to their large diffusion in IoT networks, are RFIDs, sensors, memories, and energy harvesting devices.

### 3.1. RFIDs

Connectivity to anything, the main characteristic of IoT, requires the unique addressability of things. The RFID (radio frequency identification) tag is considered a key technology for both addressing physical objects and sensing [132]. The original task of RFID is identification and tracking [133,134,135,136,137,138,139,140,141,142]. 

**Figure 4:**
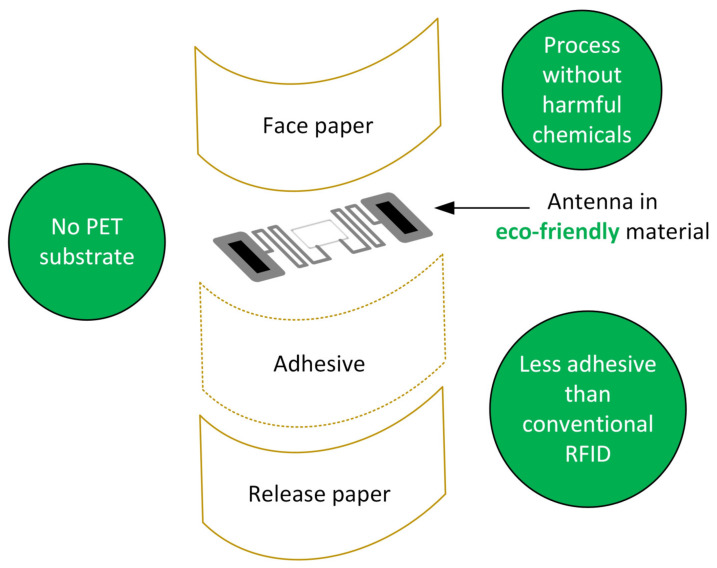
Representation of the layers that make up an eco-friendly RFID. With respect to conventional RFID, a green RFID has no plastic substrate and presents fewer adhesive materials. The production process does not involve the use of toxic or environmentally harmful chemicals.

Technological progress has made it possible to integrate sensors into the tag, thus significantly increasing and expanding the performance of RFIDs, making them ideal for use in the IoT and in sensor networks [143,144,145,146,147,148,149,150,151]. The increase of IoT and sensor networks nodes leads to a rapid and drastic growth of the number of RFID tags produced, and IDTechEx predicts that in 2023 31.841 billion passive RFID tags will be sold, which will become 41.490 billion in 2024 and 102.330 billion in 2029 [152]. RFIDs therefore constitute an important source of e-waste, and it is necessary to make their production and disposal sustainable [133,153]. Many efforts have been made, achieving excellent results, to produce eco-sustainable RFIDs, exploiting recyclable substrates (as discussed in Section 2) and eco-compatible materials for printing the antennas and for the realization of the adhesive layer [154,155,156,157,158,159,160,161,162,163,164,165]. The commitment to make the RFID technology sustainable is visible not only in the scientific literature but also in the activities of the manufacturing companies [166,167,168]. In Figure 4 a schematic representation of a green RFID is shown. With respect to conventional RFID, a green RFID has no plastic substrate and presents fewer adhesive layers. The production process does not involve the use of toxic or environmentally harmful chemicals. Table 5 reports the results obtained with eco-friendly RFID. Although the results are quite encouraging and may allow the implementation of efficient IoT nodes, a few challenges still need to be addressed. First of all, paper substrates are surely the optimum choice in terms of eco-sustainability, but their uneven surfaces and liquid absorption must be taken into consideration and possible coating processing must be evaluated and used, to improve their functionality. Secondly, conductive material for the antenna implementation should be chosen based on conductivity, mechanical deformability, and ease of adaptation to current manufacturing methods. The metal-based conductive inks are up to now the best solution, because of their good mechanical and electrical properties and printability. According to manufacturing processes, this is another fundamental issue to be addressed. The conventional etching approach for RFID tag production consumes an excess of metal materials, and requires a series of complex processing steps, resulting in a too large amount of waste material, whereas the printing technology, being an additive manufacturing process, limits the waste of conductive material and can be accomplished in few steps and at a relatively low cost. The appropriate printing method is an essential requisite for obtaining printed flexible RFID antenna patterns with good performance. 

To date, several types of printing technologies have been adopted in flexible antenna manufacturing, but lately screen printing, inkjet printing, flexographic printing, aerosol jet (AJ) and electrohydrodynamic jet (EHD) printing are preferred for their characteristics [168]. In Table 6 the key parameters of these printing technologies are reported.

Inkjet printing, which consists of transferring ink materials directly to the flexible substrates without any masks, is still the main technology to fabricate RFID, because of its simplicity. Metal solution and nano-based conductive inks are the most suitable materials for inkjet printing, because of their relatively low viscosity. The drawback of inkjet printing is that inaccuracies can affect the quality of the antenna. 

Screen printing produces a pattern by forcing the ink through a screen with a fine mesh, that is divided into graphic and non-graphical areas, defining the printed pattern. This technique is also simple to implement and accurate.

Flexo-printing is the fastest printing technology and is therefore considered when the production of very large amounts of printed antennas is required. 

AJ and EHD are the most recently developed techniques. In aerosol jet printing the ink is aerosolized and delivered to the substrate by a carrier gas to design the patterns. With respect to inkjet printing, aerosol jet printing provides a higher (up to four times) resolution, fewer strict requirements for the ink viscosity, but cannot print inks with low-boiling point solvents.

EHD printing generates very fine ink droplets by applying an electric field between the nozzle and the substrate; therefore, it is a promising candidate for high-resolution printing.

### 3.2. Sensors 

**Figure 5:**
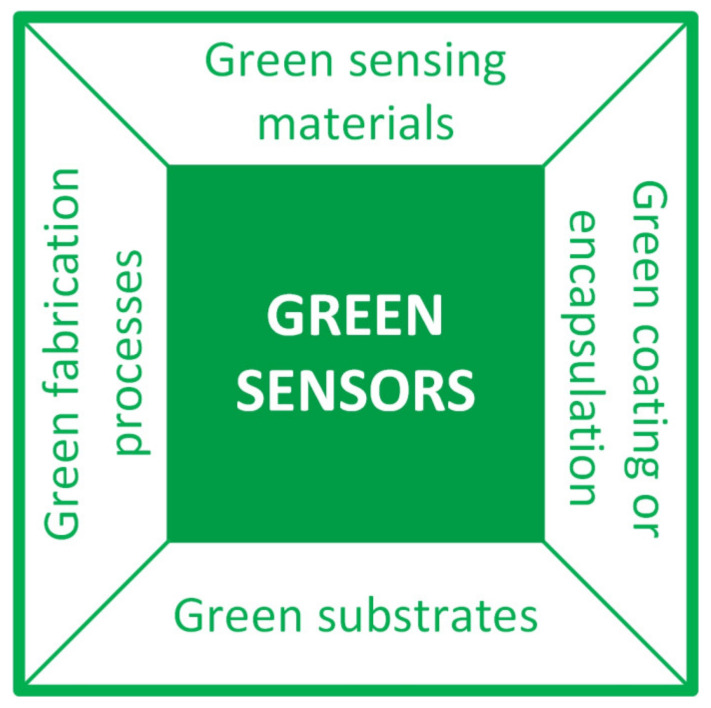
Synthetic schematization of the four fronts on which to operate to produce environmentally sustainable sensors: (1) the use of eco-friendly materials as substrates; (2) as sensing layers; (3) as coating or encapsulation; (4) the implementation of sustainable fabrication processes. For each front identified, the most current solutions based on sustainable materials and production processes are illustrated.

Undoubtedly, in all IoT systems, sensors, which are the interface with the real world, are crucial and therefore they are the main and most diffused devices of the perception layer. It is consequently essential to be able to produce green sensors and a few review papers in the scientific literature are available on this theme [169,170,171,172,173]. What emerges from these and other works is that, in order to move towards the production of green sensors, it is necessary to work on four fronts, as summarized in Figure 5: (1) the use of eco-friendly materials as substrates [174,175]; (2) as sensing layers [176,177,178,179,180,181,182]; (3) as a coating or encapsulation [175,183]; (4) the implementation of sustainable fabrication processes [184,185,186,187,188]. As far as eco-friendly substrates are concerned, those based on paper or cellulose are the most suitable. However, the strong water absorption of paper limits its use in a few applications, for example in wearable strain sensors. In these cases, a sizing agent layer that imparts hydrophobic properties to the substrate is necessary [174]. According to the sensing layers, biomaterials that feature biocompatibility, biodegradability and bioabsorbability are key solutions. As far as paper-based sensors are concerned, we refer readers to two very in-depth reviews on the subject [189,190]. In [189] Singh et al. present different types of paper that are employed in paper-based sensors, their detection mechanism and common fabrication techniques. In [190] Tai et al. illustrate the state-of-the-art of the paper-based gas, humidity, and strain sensors, offering a comparison among their characteristics and performances. For researchers interested, in particular, in the state-of-the-art of paper-based humidity sensors we recommend reading [191,192] which is a review divided into two parts that covers all types of paper-based humidity sensors, such as capacitive, resistive, impedance, fiber-optic, mass-sensitive, microwave, and RFID. Although these reviews show that considerable progress has been made on paper-based sensors, making them an interesting perspective for the sustainable future of the IoT (and beyond), they also highlight that it is undoubtedly true that some aspects still need to be explored and improved. Indeed, the manufacturing processes, the optimization of the surface and the choice of active materials must be optimized. In this regard, carbon-based materials (such as CNTs, graphite, graphene, reduced graphene oxide, graphene oxide) are currently the main sensing active layers for fabricating paper-based sensors. Considering the importance of graphene in sensor fabrication, it is worth mentioning and highlighting laser-derived graphene (LDG) technology, which is gaining attention as a promising material for the development of new electrochemical sensors and biosensors [187]. Compared to standard and well-established methods for graphene synthesis, LDG provides many advantages in terms of performance, such as fast electron mobility, good electrical conductivity, porosity, mechanical stability, and a large surface area; moreover, LDG is cost-effective and, more importantly from the point of view of environmental sustainability, it is produced by a green synthesis. To complete the overview of carbon-based sensing materials, it is also worth mentioning daily carbon ink (DCI) containing carbon black nanoparticles that, having very good performance in terms of conductivity, dispersion, adhesion, and low cost, besides a mature industrial preparation technology due to his history, could represent an excellent alternative to other materials, especially for low-cost applications. A detailed report on DCI can be found in [193]. With the advent of innovative materials, the sensing performance of the paper-based sensors is expected to further improve. However, especially with reference to novel 2D materials, challenges in the construction of high-quality 2D films on paper surfaces that are rough and porous, still need to be addressed.

Other materials that may offer environmental benefits have been explored for realizing flexible sensors for the IoT. In Table 7 we report a few examples of sensors that are interesting from an ecological point of view, indicating, where available, information on stability after bending stress. The authors of the various works reported here declare that, within the bending tests, only a slight change in the responses of the sensors was observed. 

As can be seen from Table 7, starch has been largely used as a sensing layer for flexible sensing [177,182], and [182] is an extensive review on starch applications; in [178] a sensor based on biodegradable flexible polylactic acid piezoelectric film which achieves significant longitudinal compressive and transverse tensile sensitivities and thus can act either as a pressure sensor or as a tensile sensor is described; a humidity sensor made of a thin film of electrically conductive protein nanowires is presented in [180]. In [176] an interesting solution for implementing an all-organic and all-paper pressure sensor is investigated. The use of polypyrrole (PPy) as a polymer for electrodes is one of the most popular choices in the scientific literature [206], the electrode is realized with high-conductivity PPy printing paper and the active sensing layer is implemented with low-conductivity PPy tissue paper. The structures realized with the technique reported in [176] are cuttable and foldable, therefore hollow and 3D all-paper sensors can be realized through the fabrication of kirigami or origami, granting a 3D perception capability to the sensors. The advantages of 3D structured sensors are also exploited in [183], where a skin sensor realized as a sandwich structure involving a 3D conductive network between two encapsulation layers is characterized. The sensor is biocompatible and biodegradable and encapsulated by a nontoxic water-soluble polymer. In fact, polyaniline is the active conductive filler for the 3D conductive network and silk fibroin and poly (lactic-co-glycolic acid) were used to form a network for carrying conductive materials. The 3D conductive network was encapsulated by K-carrageenan. The work presented in [202] is a novel demonstration of the combination of natural polymer (chitosan) and synthetic polymer (PVP) for next-generation semiconductor device applications.

As far as substrates are concerned, what emerged from the examined works is summarized in Table 8. From the point of view of sustainability, organic materials represent the optimal choice. Among inorganic materials, carbon materials represent a good solution. However, some magnetic materials and metals (provided they are processed as thin foils) can also offer good alternatives.

As for the materials for the sensing layer, in addition to the carbon-based materials we mentioned earlier, natural bio-origin materials possess very good features such as tailorable chemical composition as well as mechanical properties. Obviously, they are particularly attractive for sustainability, as they exhibit excellent biological characteristics such as abundant supply, biodegradability, biocompatibility, and anti-microbial activity. However, if compared to the conventional materials for electronics devices, their electrical performance is still much lower. In fact, we have found in the literature several solutions that, to overcome this limitation, involve the mixing of natural bio-origin materials with conductive materials, enabling an eco-friendly matrix for protection of the conductive components. The formed bio-composites have been shown to possess both environmental friendliness as well as high conductivity, which broadens their applications for fabricating flexible electronic devices.

Although the choice of materials is of fundamental importance to produce eco-sustainable sensors, the same importance must also be given to manufacturing techniques. With reference to this issue, in [184] the design of a green approach for synthesizing conductive polymers is discussed; in [185] a facile strategy to fabricate a compressible carbonized cellulose fiber network strengthened with in situ-synthesized polydopamine for flexible pressure sensing applications is demonstrated; in [186] a notable strategy for manufacturing a sensitive polydimethylsiloxane-derived wearable piezoresistive sensor, based on silver nanoparticles and multi-walled carbon nanotube nanocomposite films is presented that is low-cost, environmentally friendly, scalable, and industrially available. 

### 3.3. Memories

**Figure 6:**
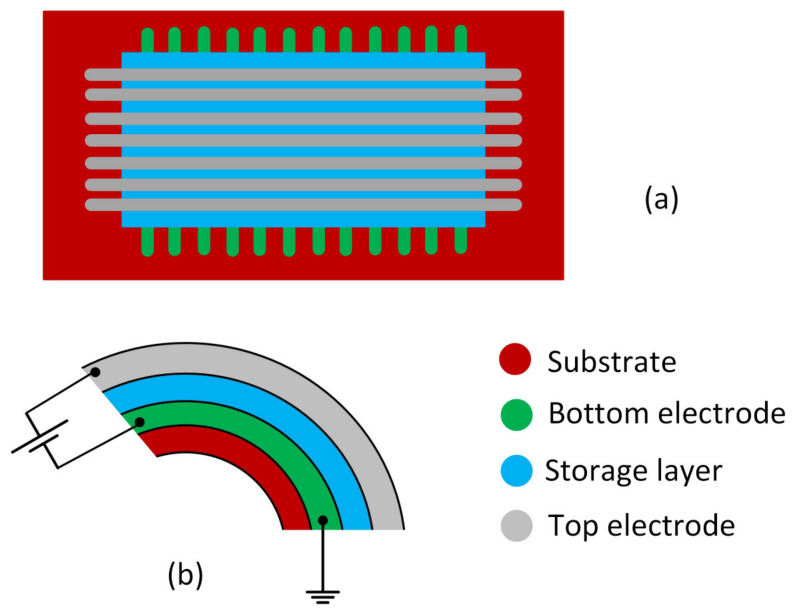
Structure of a RRAM. (**a**) RRAM top view; (**b**) flexed RRAM. Both substrate and storage layer are implemented with eco-friendly materials.

With the increasing number of nodes in IoT networks and the need for the collection of data becoming more and more common, storage devices are gaining relevance and their implementation as flexible memories is strategic in many applications [207,208,209,210,211]. Flexible resistive random access memories (RRAM) show high potential for green nonvolatile memories implementation [212]. In Figure 6 the structure of a flexible RRAM is shown, and both the substrate and the storage layer should be implemented with eco-friendly materials.

In [213] an Al/gelatin/Ag sandwiched structure on a bio-cellulose (BC) film was demonstrated, whose texture was flexible, ductile, and could be adapted to uneven surfaces. The gelatin dielectric layer and the BC substrate were non-toxic and environmentally friendly and, moreover, the BC film could be degraded completely in soil in only 5 days, thus allowing the realization of a fully biodegradable device. Biocompatible materials are used for flexible memories, such as pectin [214], that has emerged as a suitable alternative to toxic metal oxides for resistive switching applications; carbon dot-polyvinyl pyrrolidone nanocomposite and a silver nanowire (Ag NW) network buried in a flexible gelatin film [215]; starch [177,216]; poly(ethylene furanoate) (PEF), a 100% biobased polyester, as substrate, and the biopolymer deoxyribonucleic acid (DNA) as active layer [217]; iron (Fe) ions in gelatin matrixes (gelatin composites) prepared on commercially available flexible paper substrates through the solution method [218]. In [211] a poly(3,4-ethyl enedioxythiophene):polystyrene sulfonate (PEDOT:PSS)/ZnO/PEDOT:PSS transparent printed memory structure was presented that was fully fabricated using a sinter-free inkjet based process. The process conditions used in this work had the advantage of making zinc oxide non-toxic. The material selection for storage devices is particularly crucial for biomedical applications. Silk fibroin as a dielectric layer to fabricate biodegradable RRAM proved to be a good solution [219]. In [219] a W/Silk fibroin/Mg sandwich structure was studied, that provided a stable bipolar resistive switching behavior with good repeatability, surpassing the performance of most organic resistive memory and was comparable to inorganic resistive memory. Furthermore, the solubility test in phosphate buffered saline indicates the device exhibited good biodegradability. In [220] the natural biomaterial egg protein as the active layer for a RRAM was employed, and the designed device exhibited a write-once-read-many memory property.

In Table 9 the representative examples of RRAM are summarized, specifying the materials from which they were made, the performances in terms of on/off current ratio, operation voltage and data retention time, and the main benefits in terms of sustainability.

All the works reported in Table 9 declare negligible variations of the on/off ratio as a function of the bending angle and the number of cycles of bending.

### 3.4. Energy Harvesters

**Figure 7:**
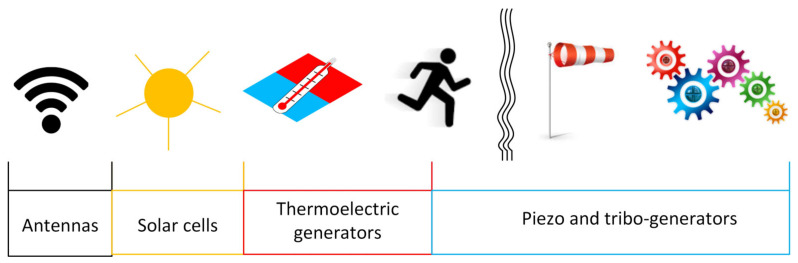
Summary of the different types of environmental energy with the relative devices used to harvest.

Considering that billions of devices will compose the IoT infrastructure in the near future, the issue of providing power supply is considered as crucial. The possibility of making each device self-powered is certainly attractive, and the energy harvesting technique is becoming more and more popular. In fact, by exploiting the energy supplied by the environment in various forms (mechanical, thermal, solar, radio frequency, wind) it is possible to replace batteries, with considerable simplification of flexible systems and, above all, with great benefits for the environment [221]. As with the other perception layer devices, the realization on eco-sustainable substrates is also essential for energy harvesting devices [222,223,224]. In Figure 7 a summary of the different types of environmental energy with the relative devices used to harvest it are shown.

The piezoelectric and triboelectric effects allow for mechanical energy harvesting, turning lost mechanical energy into valuable electrical energy [225]. Piezoelectric nanogenerators can convert the small vibrations of the environment, human body motions, etc., into useful electrical energy [226,227,228,229]; triboelectric nanogenerators provide higher output and are more cost effective [230,231,232,233]; but the combination of piezoelectric and triboelectric effects is a highly rated choice, in order to improve the output performance of a single nanogenerator, allowing the extraction of more electricity from a single device [234,235,236,237,238].

Recently, also thermoelectric (TE) energy harvesting technology using polymer-based TE materials has gained more and more attention [239]. Among the various polymers used in TE materials, cellulose plays a crucial role with a view to create devices that are not only flexible, but ecological [240]. Although organic thermoelectric materials have exhibited good performance, their thermoelectric efficiency is still too low to be commercially applied and produced and the interactions among the electric conductivity, Seebeck coefficient, and thermal conductivity still need to be optimized [241]. 

In the scientific literature there are also examples of energy harvesting that optimize performance by combining the recycling of energy from different sources. As examples, in [242] the integration of an RF energy harvester and a thermal energy harvester is presented, capable of collecting ambient energy 24 h a day; in [243] a flexible and wearable hybrid radio frequency and solar energy harvesting system for powering wearable electronic devices is discussed. In Table 10 representative examples of energy harvesting devices are reported, specifying the materials from which they are made, the type of energy they harvest, and the main benefits in terms of sustainability. All the listed solutions have proven to be a stepping-stone towards achieving self-powered, environment-friendly Internet of Things networks.

## 4. Discussion: The Perspective of the Designer

### 4.1. Designer’s Skills and Competencies

**Figure 8:**
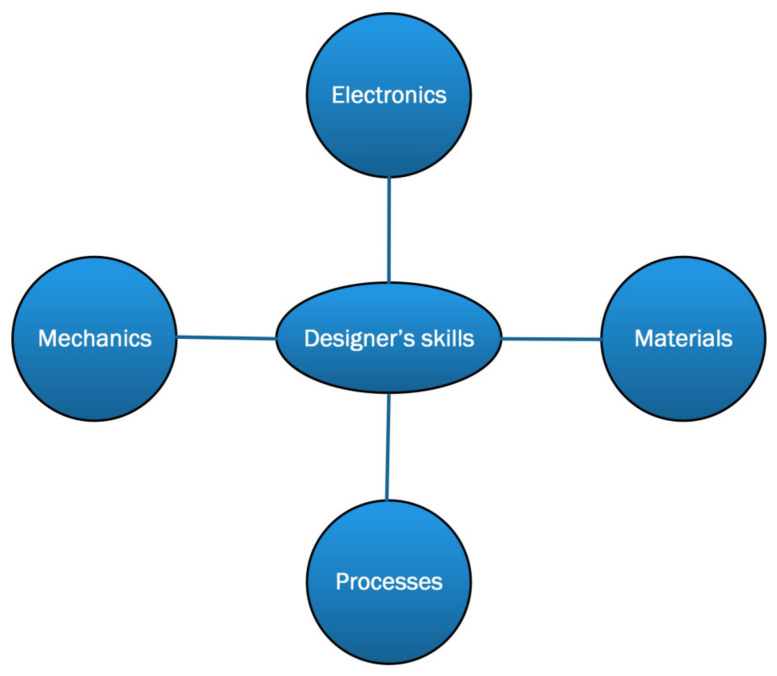
Schematization of the mandatory skills for a designer of flexible, green electronic circuits.

The need to pursue the objective of sustainability which, given the vast pervasiveness of electronic devices, requires the use of alternative materials to conventional ones (for example silicon) and power supply techniques based on energy harvesting, will inevitably change the preparation required for electronic engineers and the typical performance of simulation and design tools. To be able to design an electronic device such as those presented in the previous sections, the designer must possess not only knowledge of electronics (and device physics) but must also have a good preparation in the field of materials science in order to be able to make the correct choice of substrates and dielectric and conductive materials considering the applications and required working conditions. When dealing with flexible electronics, knowledge of mechanics and technical physics are mandatory to predict or analyze the effects of strains on the performance of devices or circuits. Additionally, if eco-sustainability is one of the project’s objectives, an in-depth knowledge of the production processes and disposal processes at the end of the devices’ life is also required. Although it is always possible to carry out a project in collaboration with researchers from different fields, to facilitate the interaction between different skills is still important to broaden one’s knowledge. The skills required in an electronic green flexible device designer are summarized in Figure 8. In addition, simulation and design tools must also integrate electronics, materials science and mechanical facilities. Therefore, the user who uses such design software must be able to handle it comprehensively.

### 4.2. Required Features of Design Tools 

**Figure 9:**
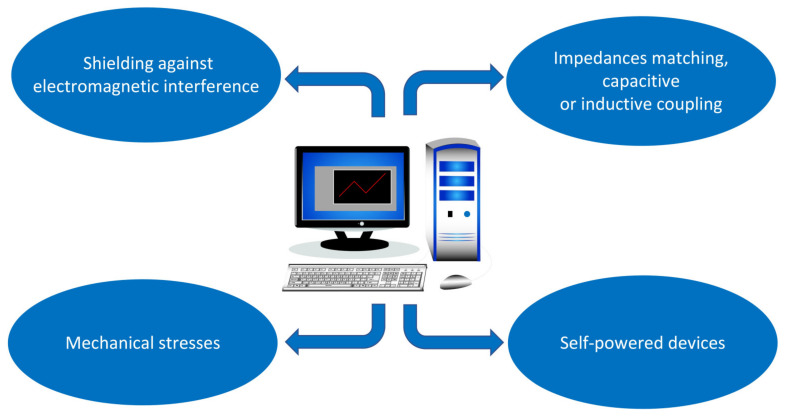
Representation of the new features that are required for modern simulation and CAD software.

Simulation tools have always been fundamental in the design of electronic circuits, and they are also fundamental to the latest generation of flexible devices. Clearly, the simulators used up until now have limitations, as they are based on models derived from conventional semiconductor theory. The design of biodegradable, eco-friendly flexible electronic devices present many challenges for electronics system simulators and CAD (computer aided design) software. For example, new tools must take into account the properties of new materials, both those used as substrates and those used as dielectrics or conductors. Their interaction with electromagnetic fields must be foreseen, by simulating the shielding capability of new polymers and biomaterials against electromagnetic interferences. Any variations in impedance, capacitive or inductive couplings, or drift of the characteristics must be considered not only as a function of the environmental working conditions, but in the presence of embedding in other materials or tissues (even humans or animals). Effects of mechanical stress (bending, stretching or twisting) on structures and electrical performances must be estimated. Given the diffusion of self-powered systems by energy harvesting techniques, simulation tools that take into account the conversion efficiency would also be useful. Based on all these considerations, a convergence between mechanical design and electronic design is desirable. In Figure 9 a summary of the new features that are required for modern simulation and CAD software is shown.

However, at present we are still far from having a simulator of this type. Progress has been made in the simulation of new materials (e.g., organic) applied to electronic devices from a physical point of view. For example, SCAPS-1D, a simulation tool for thin film solar cells developed at ELIS, University of Gent, [244] is used to better understand the physics of perovskite solar cells to optimize the devices’ efficiencies [245,246] also in conjunction with machine learning techniques [247].

Since many researchers and designers in the field of electronics usually work with SPICE-like simulators, studies aimed at importing device models implemented with new materials into SPICE are of particular importance. In [248] a compact DC model of organic thin film transistors (OTFTs) and its SPICE implementation is presented and the experimental data on the fabricated devices resulted in good agreement with SPICE simulation results; in [249] a SPICE compatible compact modeling of IGZO (Indium gallium zinc oxide) transistors and inverters having an atomic layer deposition (ALD) Al2O3 gate insulator on a flexible polyethylene terephthalate (PET) substrate is proposed, that enables a reliability-aware circuit simulation so that the operation of the flexible transistor and circuit can be predicted with high accuracy; in [250] an in-depth study of three-dimensional inkjet-printed flexible organic field-effect transistors (FETs) and integrated circuits (ICs) is reported, highlighting the necessity of modelling-driven design and analysis. With respect to such a consideration, compact modelling of the flexible printed organic FETs has been performed together with SPICE simulations of both static and dynamic behaviors of flexible printed organic circuits. This work provides insights that can fuel further improvement towards the realization of increasingly complex organic ICs and flexible electronic applications.

In addition to efforts to integrate flexible device models into SPICE, other studies have been done to simulate the mechanical deformations of devices and the related variation of electrical properties. In [251] both the biaxial and uniaxial bending stresses in polysilicon TFTs using process conditions like thermal mismatch among materials have been modeled, in [252] a framework based on the nonlinear finite element technique for obtaining stress/strain mapping from the deformation gradient for isotropic materials is developed, that in [253] has been applied to flexible electronics application. In [254] a simulation approach for evaluating the performance of arbitrarily deformed flexible electronic components is presented, that exploits a computer graphic method for three-dimensional object manipulation. The method has been validated by estimating the impact of twisting and crumpling on the performance of flexible RF antennas. 

Finally, to underline the importance of the availability of tools of simulation and CAD of advanced flexible devices, we point out that important software companies, and not just individual researchers or laboratories, are investing in this direction [255,256]. 

**Figure 10:**
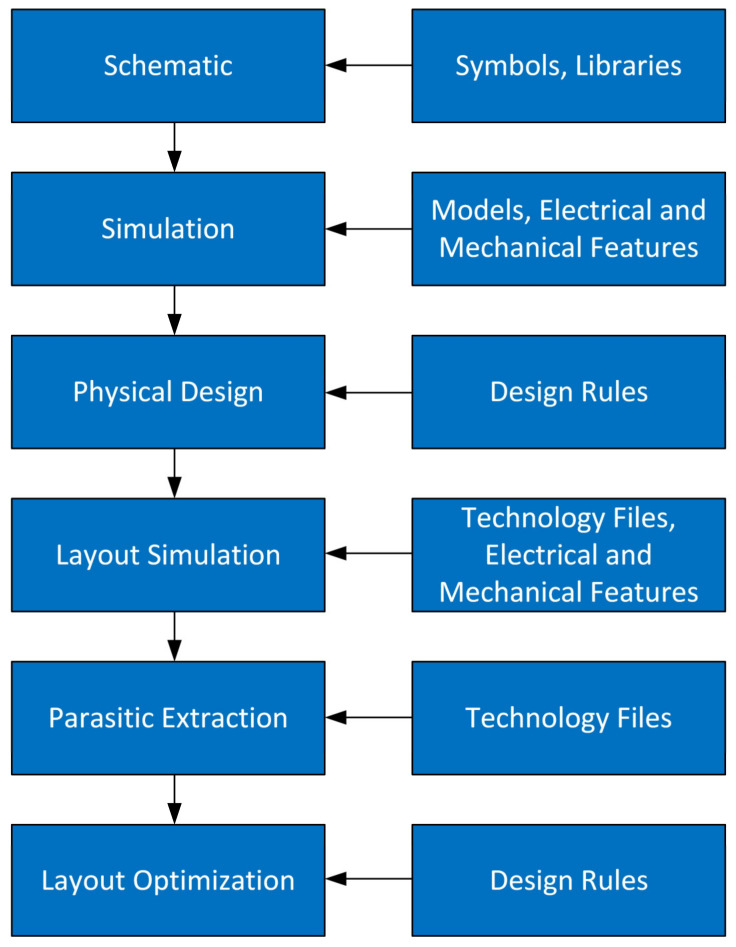
Design flux of a flexible circuit. It is not conceptually different from that of a conventional circuit, but each step is more complex.

With a simulator having all the required features available, the design flow of a flexible electronic circuit is not conceptually different from that of a conventional circuit (Figure 10). The greater complexity arises from the more numerous and varied information that each issue of the CAD software (models, technology files, libraries) must contain in order to take into consideration not only the physical and electrical aspects of the circuit, but also the mechanical ones. Furthermore, the variety of materials used for flexible electronics is certainly much wider than that of materials used for conventional electronics. We also recall that for the design flow in Figure 10 we are considering the already available and optimized substrate. If this were not the case, it would be necessary to insert a further design step aimed at a better functionalization of the substrate.

The current commercial tools [255,256] are still more aimed at a rigid-flex type design rather than a full-flex design, but provide new rules to bridge the MCAD–ECAD (mechanical CAD—electrical CAD) domains. Since reliability is the key, design rules are typically focused on the degradation of the system in the transition zone between rigid and flexible substrates and on the flexible substrate. Rules usually include the minimum bending radius, avoiding placing vias in bend areas or transition zones, avoiding placing component pads too close to the bend area, and, finally, avoiding placing stiffeners that can interfere with the bend radius and are too close to vias and pins. An inter-layer checker by means of a configurable matrix of custom DRCs (design rule checks) ensures meeting the requirements for rigid-flex designs of conductor and non-conductor layers such as soldermask, coverlay, stiffener, and adhesives. This kind of automated approach allows saving up to 50% of the project time for rigid-flex projects. Clearly, as we move towards fully flexible system design, the degree of CAD complexity will increase. First, the database of materials for flexible substrates should be extended (currently only the main plastic materials are planned) and the same for active layers. In rigid-flex design, usually the flex part of the project includes connectors, while in a fully flexible design, the core of the circuit/device is on the substrate region to be bent. Therefore, a more accurate modeling of the various layers and materials from a mechanical point of view is needed.

### 4.3. New Characterization Methods

In addition to the design method and production processes, the way of characterizing the reliability of electronic devices is also inevitably changing. 

**Figure 11:**
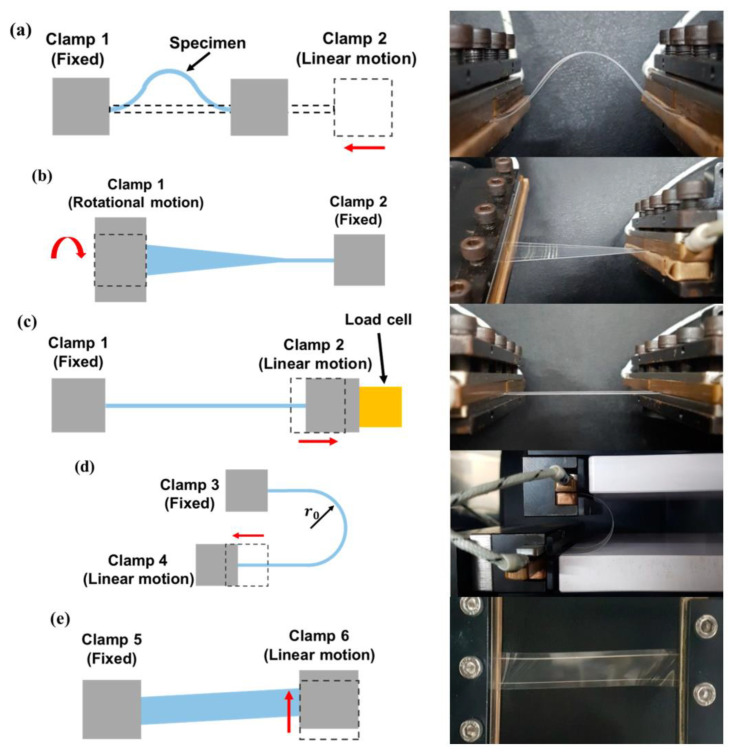
Deformation characterization of flexible devices. The image is reported from [274] under a CC BY 4.0 license. It shows the schematics and actual photographs of various deformation characterizations that are implemented by the test apparatus proposed in [274]: (**a**) linear bending mode; (**b**) twisting mode; (**c**) stretching mode; (**d**) sliding mode; (**e**) shearing mode.

First of all, a deep understanding of the charge transport mechanism of new materials (organic, biodegradable, biocompatible) is mandatory. In this regard, the technique of low-frequency noise measurements could be strategic [257]. This technique, long established for the characterization of conventional electronic devices, being highly sensitive and non-destructive, now finds application for the study of sensors, 2D materials [257], organic materials [258,259,260,261,262,263] and could therefore be used for the characterization of new flexible devices, in addition to conventional electrical characterization methods, such as current-voltage, impedance, dielectric. While usually the reliability of electronic devices is verified by electrical characterization, even under conditions of accelerated stress to obtain their lifetime, now it is also essential to carry out a mechanical characterization. In particular, in recent years, the characterization of flexible devices based on the cycles and the radius of bending has become fundamental [264,265,266,267,268] and a few researchers have dedicated themselves to the development of bending test machines [269,270,271,272]. In this regard, the technique of low-frequency noise measurements, previously mentioned as a valid tool for studying the characteristics of materials and devices, has also proven to be a sensitive tool for the characterization of the degradation of electron devices on flexible substrates as a consequence of mechanical stress [272]. Other works have also faced stretching stress [273] and various deformations tests [274] are nowadays performed to characterize the flexible samples. Figure 11, reproduced from [274], shows the schematics and actual photographs of various deformation characterizations that are implemented by the proposed test apparatus: (a) linear bending mode; (b) twisting mode; (c) stretching mode; (d) sliding mode; (e) shearing mode. Although it is only an example, Figure 10 schematizes all the mechanical stresses that the flexible circuits should be subjected to for a complete performance evaluation. Although devices with this potential are not yet widespread, it would be a good solution to perform both electrical and mechanical characterization simultaneously, to better understand the correlation between mechanical stress and electrical properties and to monitor eventual changes in real time. Much work still needs to be done in this field, to perfect the various techniques and make them available in all research laboratories.

## 5. Conclusions

In this brief review, the main solutions of flexible devices for IoT applications that are fabricated with the goal of environment sustainability are reviewed. RFID, sensors, memories and energy harvesters implemented in biodegradable materials, with eco-friendly structures and fabricated with environmentally sustainable processes are described. The first evident limitation of the analyzed technologies is that, except for some very rare examples in the literature, they are not yet able to be integrated and to supply more complex flexible electronic systems. The production of flexible electronics is therefore still limited, almost exclusively, to the manufacture of single devices or rather small and simple systems. Much research still needs to be done in this direction, also evaluating the possibility of resorting to mature and effective technologies in industry that have driven the development of traditional rigid semiconductor devices and that can also be potentially applicable to flexible devices. The integration of flexible technologies with ultra-thin chips based on silicon, the semiconductor with the best performances in electronics, would combine flexibility and scalability (for sensing, actuating, energy harvesting) of the new flexible devices with the high efficiency of silicon for computation and communication purposes. However, although this way would certainly lead to very high-performance systems, integration with silicon would slow down the achievement of the goals in terms of environmental sustainability, also considering the ever-increasing number of nodes within an IoT network and the number of networks itself. According to the materials, both organic and inorganic materials are promising for sustainable flexible electronics, both for substrates and active layers. However, further research and development are required to improve stability, functionality, and both electrical and mechanical performances. Manufacturing technologies, and above all printing technologies, still need to be optimized, especially as currently process variability remains a challenge. The simplicity of disassembly processes in the end-of-life phase of the devices must also be evaluated for the choice of materials, in order to guarantee the possibility of recycling or biodegradability. Finally, the skills required for designers of electronic circuits are changing, as they must have competences not only on the electrical properties of materials and the physics of conventional devices, but also on materials science and mechanics. Even the simulation and design tools for electronic circuits must include features dedicated to the design of flexible circuits with organic materials and the characterization of the realized devices must be performed also considering mechanical stresses. At present, there is not yet a professional figure who has all the necessary skills, therefore it is necessary that the work in this field is carried out by research groups from different scientific fields. Furthermore, although some CAD software manufacturers are adapting to the new demands, there are still no tools largely available similar to those for conventional circuit design. The same goes for the equipment needed for the characterization of new flexible electronic systems, for which researchers almost always have to resort to self-made instrumentation and equipment.

## Figures and Tables

**Table 1:** Comparison between paper and the most used plastic substrates, in terms of the impact on climate change and resource use.

Substrate Material	Climate Change Impact kg CO_2_ eq. */Sheet ***	Resource Use kg Sb eq. **/Sheet ***
Paper	1.3 × 10^−4^	5.2 × 10^−11^
PET (polyethylene terephthalate)	1.5 × 10^−3^	1.8 × 10^−10^
PEI (polyetherimide)	1.3 × 10^−2^	2.0 × 10^−9^
PEEK (polyether ether ketone)	7.4 × 10^−3^	2.2 × 10^−9^

* Indicator of potential global warming due to emissions of greenhouse gases to the air. ** Indicator of the depletion of natural non-fossil resources. *** Sheet with 25 cm^2^ surface area, 125 mm thickness.

**Table 2:** Comparison between conductivity of the printed layer on paper and PET substrates. The layer thickness used in the volume resistivity measurement was considered to be equal on every substrate. An ink transfer volume of 7 mL/m^2^ has been considered.

Printing Technique	Substrate Material	Sheet Resistance mΩ/Square	Volume Resistivity (Ω·cm)
Flexo-printing	P1 *	177 ± 19	2.2 × 10^−6^
	P2 **	169 ± 16	1.6 × 10^−6^
	PET ***	260 ± 23	2.1 × 10^−6^
Rotary screen-printing	P1	45.3 ± 1.3	4.1 × 10^−5^
	P2	39.4 ± 0.6	3.4 × 10^−5^
	PET	52.3 ± 2.5	4.7 × 10^−5^

* Coated paper, Stora Enso NovaPress Silk, 80 g/m^2^. ** Coated paper, ultra-smooth top side for printed electronics, Arjo Wiggins PowerCoat HD, 95 g/m^2^. *** Melinex ST506 (DuPont Teijin Films, Chester, VA, USA).

**Table 3:** Comparison of Young’s modulus of paper and plastic substrates [109].

Substrate Material	Young’s Modulus [GPa]
Paper	Up to 3.5 *
PET	2.8
PEN	3.0
PDMS	Up to 3.7 **

* Depending on coating. ** Depending on different crosslinking density.

**Table 4:** Example of biodegradability test on cellulose based and plastic samples. The test duration was 127 days [114].

Sample *	Status	Biodegradation **
CNF 50%, HEC 50%	Printed	74%
CNF 50%, HEC 50%	Unprinted	78%
MCC	Unprinted	94%
TPU	Unprinted	No degradation

* CNF: cellulose nanofibrils; HEC: hydroxyethyl cellulose; MCC: microcrystalline cellulose; TPU: thermoplastic polyurethane. ** The data are extrapolated from [114]. Biodegradation of samples was estimated firstly by employing the CO_2_ evolution method and, secondly, by visually evaluating samples disintegration in soil upon burial.

**Table 5:** Characteristics and performances of eco-friendly RFIDs. All the reported examples operate in the UHF (ultra-high frequency) band.

Material	Dimensions (mm^2^)	Gain (dBi)	Reading Range (m)	Ref.
Paper substrate	63.6 × 25	2.37	-	[155]
Copper ink on paper dielectric substrate	81.95 × 14.5	1.81	-	[156]
Graphene ink on paper substrate	16 × 65	−5	3.5	[157]
Bioresorbable copper-based paint on a bioresorbable cellulose-based substrate	79 × 8	−0.5	10.2–12.7	[159]
Sustainable conductive ink on cellulose-based substrate	36 × 120	1.7	-	[161]
Paper substrate	101.2 × 10.5	2.75	6.88	[162]
Paper substrate	92.4 × 10	3.1	9.22	[162]

**Table 6:** Key parameters of most used RFID printing technologies [168].

Technology	Ink Viscosity (cP)	Line Width (μm)	Layer Thickness (μm)	Speed (m/s)
Inkjet	10–30	30–50	1	Slow
Flexo	50–500	50–100	<1	~8
Screen	500–5000	30–50	5–100	~1
EHD	1–15,000	0.1	<1	slow
AJ	1–1000	5	<1	slow

**Table 7:** Examples of sustainable sensors for IoT.

Sensing	Material	Main Characteristics	Bending Cycles	Ref.
**Strain**		**Gauge Factor**		
	Starch, porous carbon	134.2	>1000	[194]
	Paper/MXene/sizing agent (PMS)	161 (bending angle of 0–120°)	100,000 (bending deformation of 30°)	[174]
	Starch, egg white, Ag	-	>1000	[195]
	Candle soot (CS) particles, chitosan, potato starch (PS), polyvinyl alcohol (PVA), Fe3+ ions	1.49 at 0 to 60% strain; 2.71 at 60–100% strain	>1000	[196]
	Graphite powder and cellulose fibers from waste printing papers	27	1000	[197]
**Pressure**		**Pressure range and/or sensitivity**		
	Polylactic acid piezoelectric film (DS-PLA)	0.03–62 kPa	1.08 million at a pressure of 4.3 kPa	[178]
	Starch, porous carbon	0–250 kPa	>1000	[194]
	Poly(ether carbonate)-based Polyurethane	0.62–62.5 kPa	6250	[198]
	PPy, paper	4.8 kPa^−1^ at < 5.5 kPa, 1.7 kPa^−1^ at 5.5–40 kPa	3D	[176]
	Polyaniline, silk fibroin, poly (lactic-co-glycolic acid), K-carrageenan	165.3 kPa, 2.54 kPa^−1^	>2000	[183]
	PDA–CCFN	0 to 50 kPa	1000, by repeatedly loading and unloading a pressure of 20 kPa	[185]
	AgCNT@textile-PDMS	0.02 kPa^−1^ and 0.004 kPa^−1^ in the low-pressure (<11.67 kPa) and high-pressure (~11.67–33.3 kPa)	-	[186]
**Humidity**		**RH linear range and/or sensitivity**		
	PEDOT:PSS electrode, CNF film	20% to 85%RH	-	[199]
	Graphene inks	30%RH to 90%RH linear range; 0.55/%RH at 25 °C	-	[179]
	E-PNs, *G. sulfurreducens*, Au electrodes, PI substrate	20% to 95%RH; >6% relative conductance change per 1% RH change	>1000 bending cycles	[179]
	Cellulose nanofiber/graphene nanoplatelet	30%RH to 90%RH	>1000 bending cycles	[200]
	Common kitchen salt (NaCl)	40% RH up to 85% RH	-	[201]
		**Sensitivity**		
**H_2_O_2_**, **glucose**	BGC printed inks	27.25 μA mM^−1^ cm^−2^	70 cycles by 10% stretching and 1800 consecutive 90° bending cycles	[180]
**H_2_ gas**	Chitosan/polyvinylpyrrolidone (CHP) polymeric substrate; ZnO thin film	24% and 46% towards 0.5% and 2% H_2_	-	[202]
**NO_2_ gas**	Dried mango peel, graphene, ZnO and carbon nanotubes	ΔR/R0 = 0.21 at 100 ppm and RH = 35%	300	[203]
**CO_2_ gas**	p(D-co-M)	10^4^–10^6^ ppm detection range	-	[204]
**NH_3_**	Cotton fibers/polyaniline (PANI)	100 ppm NH_3_	Stability in bending from 0° to 60°	[205]

**Table 8:** Principal substrate materials for sustainable flexible IoT.

Substrate	Materials	Biodegradable	Recyclable
Inorganic Materials	Carbon	Yes	Yes
	Magnetic	Yes, except for ceramics	Not always, but reusable
	Metals (thin foils)	Mo, Fe, W, or Zn	Not always, but reusable
Organic Materials	Polymers	Yes	Yes
	Textiles	Yes	Yes
	Silk	Yes	Yes
	Paper and cellulose-based	Yes	Yes

**Table 9:** Representative examples of flexible, eco-friendly RRAM.

Material	On/Off Current Ratio	Operation Voltage (V)	Data Retention Time (s)	Pros for Sustainability	Ref.
Al/gelatin/Ag sandwiched structure on a bio-cellulose film	>10^4^	<3	7 × 10^3^	Fully biodegradable device	[213]
Ag/pectin/FTO	10^4^	<3	10^8^	Biocompatibility derived from use of natural pectin	[214]
Carbon dot (CD)-polyvinyl pyrrolidone (PVP) nanocomposite and a silver nanowire (Ag NW) network buried in a flexible gelatin film	>10^2^	−1.12	>10^4^	Fully biocompatible	[215]
Au/starch/ITO/PET	10^3^	0.25	10^3^	Biocompatible materials	[216]
Au/starch–chitosan/ITO/PET	100	0.25	10^4^	Biocompatible materials	[216]
Poly(ethylene furanoate) (PEF) as substrate; deoxyribonucleic acid (DNA) as active layer	10^4^	−2	10^4^	Biomaterials	[217]
Iron (Fe) ions in gelatin matrixes on paper substrates	10^5^	<4.2	7 × 10^4^	Gelatin materials are biodegradable and recyclable	[218]
W/silk fibroin/Mg	10^5^	2.0	-	Good biodegradability	[219]
Egg protein and graphene quantum dot composites	1.19 × 10^4^	0.3	10^4^	Good biodegradability	[220]

**Table 10:** Representative examples of flexible energy harvesting devices.

Energy Source	Type of EH	Material	Output ***	Pros for Sustainability	Ref.
Body motion	PENG *	ZnO nanorods on the surface of paper	V_o_ = 15 mV; I_o_ = 10 nA	Cost-effective; paper substrate	[226]
Mechanical	PENG	Lead-free organic inorganic hybrid perovskite	V_o_ = 94.5 V_pp_, I_o_ = 19.1 μA_pp_; output power density of 18.95 μW/cm^2^	Lead-free	[227]
Body motion	PENG	ZnO	V_o_ = 15 mV	Eco-friendly, low temperature and low-cost process	[228]
Body motion	TENG **	Worn-out textiles from the waste bin	V_o_ = 4.2 V; I_o_ = 2.7 nA	Promote the eco-friendly concept of recycling, reuse	[230]
Wind	TENG	Rabbit fur	For wind speed of 6 m/s, peak power = 11.9 mW; conversion efficiency of 15.4%	Smart-farming applications without environment deterioration	[232]
Mechanical	TENG	Polyvinyl butyral (PVB); indium oxide (IO); Mylar	V_o_ = 700 V; I_o_ = 1.52 mA/m^2^	Energy-saving	[233]
Wind, Mechanical	TENG	Natural leaf as an electrification layer and electrode	P ≈45 mW m^−2^	Natural materials	[232]
Wind	TENG	Plant leaf and leaf powder	I_o_ = 60 μA; V_o_ = 1000 V	Natural materials	[232]
Vibrational	TENG	Cellulose acetate nanofibers (CANF) and micro-patterned PDMS	V_o_ = 400 V; I_o_ = mA/m^2^	Cellulose-based; biocompatible and biodegradable material	[237]
Mechanical	PTENG	MoS2-PVDF	V_o_ = 35.3 V	Energy saving for smart wearable devices	[234]
Biomechanical	PTENG	Bi4Ti3O12 (BiTO)/polydimethylsiloxane (PDMS)	V_o_ = 300 V; I_o_ = 4.7 μA	Simple and cost-effective fabrication technique	[235]
Hand clapping	PTENG	PVDF; Textured PDMS and skin	V_o_ = 750 V, I_o_ = 400 μA	Human skin-based; can promote additional health benefit for people	[238]
Waste heat energy	Thermoelectric	Silicon rubber sheet, electrodeposited n-type thermoelectric material	V_o_ = 1 V under a temperature difference T of 60 °C.	Mountable on complex geometries for powering wireless IoT sensing systems in smart agriculture, smart home, industry application, and environment monitoring	[239]
Thermal	Thermoelectric	Origami and kirigami-enabled resorbable TE paper, with a self-assembled inorganic particle network layer below the cellulose polymer bio-matrix layer	V_o_ = 38.55 mV, I_o_ = 12.14 μA for a temperature difference of 24 K	Significant implications in the field of green technology; completely decomposed without carbon emission in water	[240]
RF and solar	Rectenna; solar cell	Ti and Au on PDMS; amorphous silicon	2613.6 μW in sunny outdoor. Additional 3.3–37.5% hybrid output dc-power when the RF source power is varied from 9 to 14 dBm	Energy saving: high efficiency	[243]

* PENG: Piezoelectric Nanogenerator. ** TENG: Triboelectric Nanogenerator. *** Output voltage (V_o_), output current (I_o_), output power or conversion efficiency are reported are reported according to the availability of data in the references.

## Data Availability

Data sharing not applicable.
